# Pharmacological Predictors of Antidepressant-Associated Mania/Hypomania: Findings from a Nationwide Greek Cohort Study

**DOI:** 10.1192/j.eurpsy.2026.10191

**Published:** 2026-07-31

**Authors:** V. Karageorgiou, I. Michopoulos, P. Mitrou, E. Thireos, K. Mathioudakis, P. Skapinakis, R. Gournellis

**Affiliations:** 1 National and Kapodistrian University of Athens; 2 Hellenic Ministry of Health; 3 National Agency for Quality Assurance in Health S.A., Athens, Greece; 4 IDIKA SA - e-Government Center for Social Security Services, Athens; 5 University of Ioannina, Ioannina, Greece

## Abstract

**Introduction:**

Antidepressants (ADs) are widely prescribed for a variety of depressive disorders, but may precipitate manic episodes (antidepressant-associated mania or hypomania, AAMH). Identifying predictors and rates of AAMH in real-world settings is crucial to stratify clinical risk, especially in the absence of prophylactic mood stabilizers.

**Objectives:**

We aim to identify AD that carry the highest risk of AAMH via a triangulation of evidence framework that combines nationwide prescription datasets, large pharmacovigilance databases and genetics-informed pharmacodynamic data.

**Methods:**

We analyzed the Electronic Governance of Social Security (IDIKA) database encompassing patients with diagnoses of unipolar and manic episodes/bipolar depression. Incident AD users were identified based on a minimum of six observed months with no recorded AD prescriptions. Patients with prior manic or bipolar diagnoses were excluded. AAMH was defined as a new manic diagnosis within a maximum of 180 days post-AD initiation. Cox proportional hazards models stratified by AD were used to evaluate predictors of switch. The FDA Adverse Event Reporting System database was used for the extraction of manic or hypomanic episodes that follow an AD prescription. To further substantiate mechanistically any observed differences in those two sources, publicly available pharmacodynamic data on the affinities of each drug were used and corresponding variants were assessed for their strength of association with various mood-related phenotypes.

**Results:**

Among incident AD users without prior recorded mania (n=134,353, mean (SD) age: 64 (17) years, 66% female), a small proportion experienced manic switch during follow-up (992 events, switch rate = 0.7%). Adjusted logistic and survival models revealed heterogeneity in risk between ADs. Venlafaxine (adjusted hazard ratio (aHR) 1.74, 95% CI: 1.37–2.22, p<10⁻⁶) and mirtazapine (1.65, 95% CI: 1.28–2.12, p=9×10⁻⁵) had the highest rates, whereas citalopram (1.04, 95% CI: 0.78–1.40, p=0.69) and escitalopram (baseline switch rate 0.58%) had the lowest. Moclobemide, although rarely prescribed, was a notable outlier (aHR 21.1, 95% CI: 6.7–66.3, p<10⁻⁶). Age and female gender were modest predictors, with higher risk in younger female patients. The FAERS database showed largely concordant results, with vortioxetine ranking first and citalopram last. Few differences were observed with respect to mapping of the drug target genes to mood-related phenotypes.

**Conclusions:**

There are important differences in the AAMH risk profile of ADs. While overall switch rates were low, notable differences emerged by drug class, with monoamine oxidase inhibitors, SNRIs, and mirtazapine showing high rates. The largest predictor was prior BD history. Although absolute risks were lower than prior evidence suggests, clinicians should be cautious of prescribing higher-risk ADs in individuals with latent bipolarity.

**Disclosure of Interest:**

None Declared

